# The therapeutic potential of high-dose inhaled nitric oxide for antimicrobial effects: a narrative review and future directions

**DOI:** 10.1186/s40635-026-00852-1

**Published:** 2026-02-05

**Authors:** Lorenzo Berra, Nikolay Kamenshchikov, Asher Tal, Bijan Safaee Fakhr, Emanuele Rezoagli, Rachel Thomson, Binglan Yu, the High-Dose Inhaled Nitric Oxide Investigators, Lorenzo Berra, Nikolay Kamenshchikov, Asher Tal, Bijan Safaee Fakhr, Emanuele Rezoagli, Rachel Thomson, Amir Elalem, Huajie Li, Bin Wang, Run Dong, Elisa Mereto, Andrea Bolchini, Matthew Ludwig, Thomas Lambert, Cristina Miett, Stefano Spina, Hatus Wanderley, Ryan Carroll, Giovanni Bruno

**Affiliations:** 1 Department of Anesthesia, Critical Care and Pain Medicine, Anesthesia Center for Critical Care Research, Massachusetts General Hospital, 55 Fruit Street, Thier Research Building Room#505, Boston, MA 02114 US; 2 Harvard Medical School, Massachusetts General Hospital, Boston, MA US; 3 Respiratory Care Services, Massachusetts General Hospital, Boston, MA US; 4 Cardiology Research Institute, Tomsk National Research Medical Center, Russian Academy of Sciences, 111a Kievskaya St, Tomsk, 634012 Russia; 5 Faculty of Health Sciences, Ben-Gurion University of the Negev, Be’er Sheva, Israel; 6 Anesthesiology and Intensive Care, Fondazione IRCCS San Gerardo Dei Tintori, Monza, Italy; 7 School of Medicine and Surgery, University of Milano-Bicocca, Monza, Italy; 8 Greenslopes Clinical School, Faculty of Health, Medicine and Behavioural Sciences, The University of Queensland, Brisbane, Australia; 9 Gallipoli Medical Research, Greenslopes Private Hospital, Brisbane, Australia; 10 Division of Pediatric Critical Care Medicine, Massachusetts General Hospital for Children, Boston, MA US

**Keywords:** Nitric oxide, Nitrogen dioxide, Methemoglobin, Pneumonia, Multidrug-resistant pathogens, Antimicrobial therapy

## Abstract

Inhaled nitric oxide (iNO), long used as a selective pulmonary vasodilator, has demonstrated potential antimicrobial and antiviral properties when administered at high concentrations (> 20 parts per million, ppm). While definitive evidence is still lacking, this narrative review synthesizes the emerging clinical and mechanistic properties supporting high-dose iNO as a potential therapeutic strategy for lower respiratory tract infections, including drug-resistant bacterial pneumonias, COVID-19, nontuberculous mycobacteria, and bronchiolitis. We summarize safety data from laboratory studies, Phase I trials, clinical findings from 27 predominantly early-phase studies, and highlight its as both hospital-based and home-based therapy. High-dose iNO acts through multiple pathways, including direct microbial killing, biofilm disruption, immune modulation, and mucociliary enhancement, and holds promise in addressing unmet needs in respiratory infection management. We also propose a roadmap for future research to optimize dosing, delivery, and efficacy endpoints in well-defined patient populations.

## Take-home message

High-dose inhaled nitric oxide is a potential antimicrobial therapy with broad-spectrum activity against bacteria, viruses, fungi, and parasites, and has been safely administered in diverse clinical contexts from ICU to outpatient care. This review summarizes translational and early clinical data and outlines a roadmap for future trials needed to define safety, efficacy, and optimal use in drug-resistant lung infections and acute respiratory failure.

## Introduction

Nitric oxide (NO) is a potent antimicrobial molecule endogenously produced by immune cells, particularly macrophages, which generate NO and reactive nitrogen species to combat a wide array of pathogens [1–7]. Since the early 1980s, foundational studies have established NO’s critical role in innate immune defense.

**Fig. 1:**
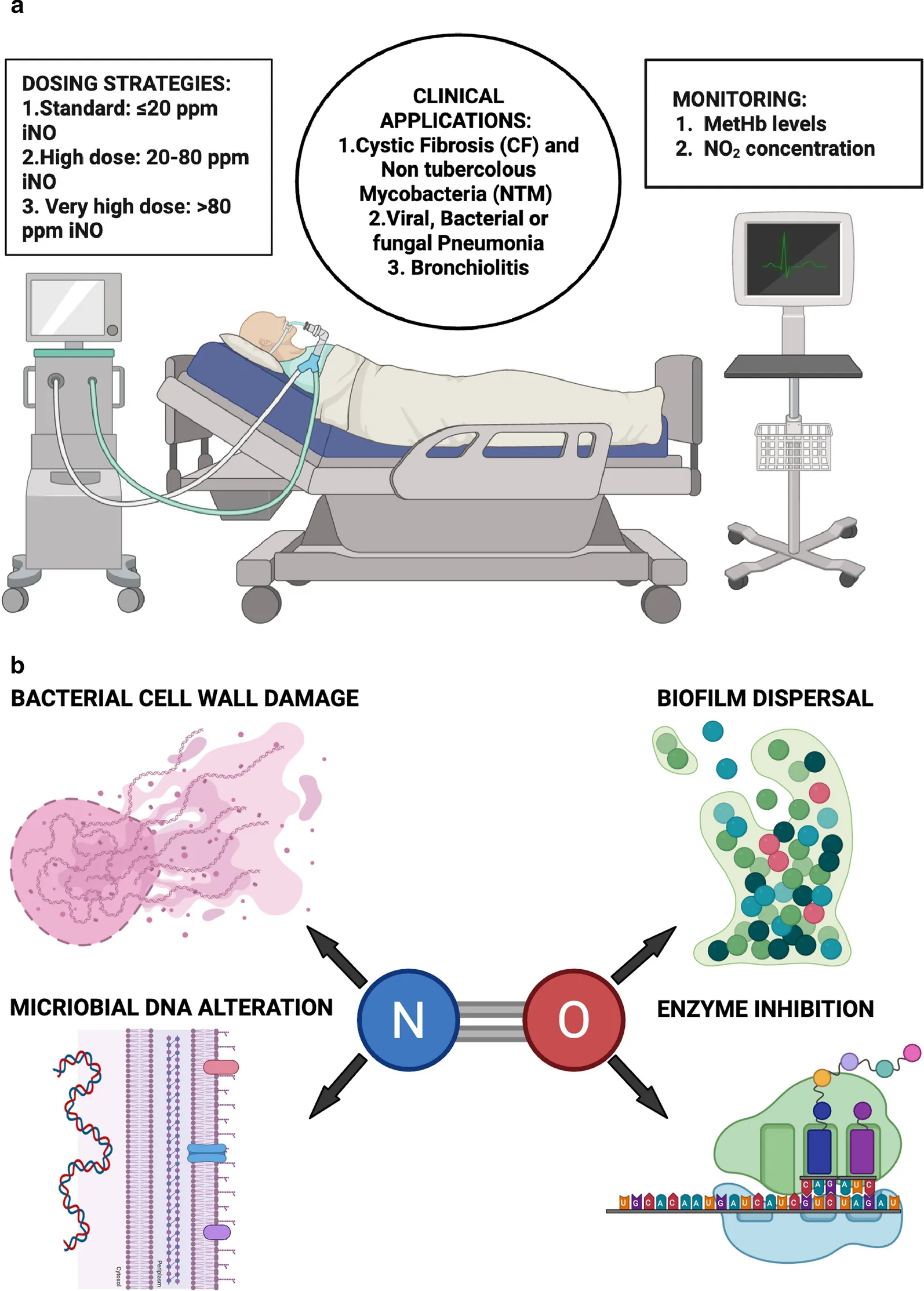
High-dose inhaled nitric oxide as antimicrobial therapy. Sketch on mechanisms of action, dosing strategies, clinical applications and safety considerations on the therapeutic use of high-dose inhaled nitric oxide

In 1991, two anesthesiologists, Claes Frostell and Warren Zapol pioneered the administration of inhaled NO (iNO) to treat pulmonary hypertension in lambs, a breakthrough that laid the foundation for its clinical use [8]. These seminal findings led to the U.S. Food and Drug Administration’s (FDA) approval of iNO at a dose of 20 parts per million (ppm) for the treatment of hypoxic respiratory failure associated with pulmonary hypertension in term and near-term neonates (≥ 34 weeks gestation) [9, 10]. Beyond its established vasodilatory role [11], iNO has recently been investigated as a potential antimicrobial therapy, particularly for lower respiratory tract infections [12]. Antimicrobial mechanisms of NO have been extensively studied [13–15]. NO is an unstable molecule that, in the presence of oxygen, can form a variety of intermediates including peroxynitrite, nitrogen dioxide (NO_2_), S-nitrosothiols, dinitrogen trioxide and, in the presence of iron, dinitrosyl iron complexes. It exhibits selective toxicity, meaning it preferentially harms invading microorganisms while minimizing damage to host cells. NO can modulate the antimicrobial immune response through several mechanisms, including (I) ubiquitous nitrosation of proteins, impairing their functions [16, 17]; (II) modification of the DNA sequence resulting from deamination of deoxyribonucleotides [18–21]; (III) induction of lipid peroxidation impairing cell wall integrity [22, 23]; and (IV) disruption of iron–sulfur clusters of the respiratory chain [24] (Fig. 1).

According to the 2024 Global Burden of Disease Report, pneumonia was responsible for over 2 million deaths globally, disproportionately affecting children under five and adults over 70, who accounted for more than 500,000 and 1 million deaths, respectively [25]. Moreover, antimicrobial resistance (AMR) represents a parallel and escalating crisis, contributing to an estimated 4.71 million deaths annually, with 1.14 million directly attributed to drug-resistant infections [25]. The most frequently implicated pathogens include *Escherichia (E.) coli*, *Staphylococcus aureus*, *Klebsiella pneumoniae*, *Streptococcus pneumoniae*, *Acinetobacter baumannii*, and *Pseudomonas (P.) aeruginosa*.

In this context, the antimicrobial properties of iNO represent a promising but still exploratory avenue for innovation. Much of the pioneering work in high-dose iNO feasibility originates from the authors’ affiliated hospital and institution—Massachusetts General Hospital, Harvard Medical School (Boston, MA). This review summarizes evidence from 27 clinical studies exploring the administration of iNO at doses higher than the FDA-approved 20 ppm. These investigations suggest that higher-dose iNO may reduce the burden of pneumonia, both community-acquired pneumonia (CAP) and hospital-acquired pneumonia (HAP), and infections caused by multi-drug resistant (MDR) organisms. High-dose iNO may also play a role in more chronic infections (e.g., nontuberculous mycobacteria) in the setting of bronchiectasis and cystic fibrosis (CF).

### High-dose iNO in healthy subjects and phase l clinical trials

The FDA has identified four warnings and precautions regarding the use of iNO: hypoxemia due to methemoglobinemia (MetHb), airway injury from nitrogen dioxide (NO_2_), rebound pulmonary hypertension following abrupt discontinuation, and worsening heart failure [26]. Since iNO is commonly used in cardiac surgery, it is important to underline that iNO can worsen left ventricular failure (LVF) by increasing left ventricular filling pressure [27]. In patients with LVF, the decrease in pulmonary vascular resistance (PVR) from iNO can be associated with an increase in pulmonary capillary wedge pressure (PCWP) and left ventricular end-diastolic pressure (LVEDP), leading to a backup of fluid into the lungs and potentially causing pulmonary edema. Three studies have been conducted to evaluate the safety of high-concentration iNO in relation to these concerns and to assess potential injury to other organs (Table 1) [28–30].

**Table 1:** High-dose iNO in healthy individuals/Phase l studies

Study	Study design	Patients	NO dose and source	Safety endpoint	Adverse event	Outcome
Miller et al. 2012	Phase I	10 healthy individuals	160 ppm × 30 min × 5/day for 5 days	MetHb < 5% NO₂ < 5 ppm SpO₂ > 85% Significant BP, RR, and HR changes Adverse events noted	2 subjects: transient tongue numbness during NO treatment	160 ppm iNO well tolerated No effect on lung function or inflammatory markers Favorable safety profile, no major adverse effects
			An INOmax, 800 ppm pressurized cylinder, Ikaria, USA			
Gianni et al. 2022	Phase I	12 healthy individuals	160 ppm × 15 min × 2/day for 14 days	MetHb < 5% NO₂ < 3 ppm SpO₂ > 90% HR changes Adverse effects occurred	0 subjects: reported discomfort, cough, wheezing, or other adverse events during NO administration	High-dose iNO delivered via cylinders and eNO generators No significant adverse effects Both methods were safe and effective
			An 850 ppm NO/N_2_ cylinder from Airgas, USA An eNO generator (Odic, USA) producing 1180 ppm NO from air via pulsed electrical discharge			
Yu et al. 2025	Phase I	10 healthy individuals	300 ppm × 30 min × 3/day for 5 days	MetHb < 10% NO_2_ < 5 ppm	1 subject: mask discomfort	NO₂ < 5 ppm throughout MetHb < 10% (peak 2–3%) No serious adverse events; mild symptoms transient and self-resolving
			A 20,000 ppm NO in N₂ cylinder was used (Airgas, USA)			

The first Phase I trial in healthy subjects, Miller et al*.* suggests that MetHb levels increased an average of 0.9% during inhalation of 160 ppm of NO and returned to pre-treatment values after discontinuation of iNO therapy [28]. The highest level of MetHb measured by non-invasive co-oximetry was 2.5%. The highest level of NO₂ recorded was 2.8 ppm, with a mean of 2.3 ppm. Later, Gianni et al*.* [29] extended these findings by testing 160 ppm of iNO as a preventive measure against COVID-19 in healthcare workers while comparing pressurized NO cylinders and electrical NO (eNO) generators. Both methods (171 via iNO tanks, 14 via eNO generator) delivered NO safely, with no adverse events. MetHb increased from 0.85% to 1.98%. Five minutes after stopping NO administration, MetHb decreased to 1.87%. NO_2_ concentrations varied from 0.70 to 0.88 ppm. Oxygen saturation (SpO₂) was stable between 96 and 97%, and the hemodynamics were unchanged. The latest study by Yu et al*.* [30] enrolled ten healthy adult volunteers who received iNO at a concentration of 300 ppm for 30 min, three times daily over five consecutive days (total of 15 treatments). NO_2_ averaged between 1.0 and 2.1 ppm depending on the sampling sites (at mouth or in the inspiratory limb), and MetHb peaked to 9.0 ± 1.1% (range: 6.8–11.3%) during exercise on day 5. In addition to non-invasive, continuous MetHb monitoring using a pulse co-oximeter (Masimo), we also collected blood samples before and after iNO treatment on days 1 and 5 to measure MetHb level with a blood gas analyzer (ABL90 Flex, Radiometer America, Inc.). The MetHb concentrations obtained from the blood gas analyzer, peaked at 6.7 ± 1.3% (range: 3.9–8.0%). None of the healthy subjects had anemia, with an average hemoglobin level of 13.9 ± 1.5 g/dL. The MetHb values measured with the pulse co-oximeter (9.0 ± 1.1% with a range of 6.8–11.3%) were higher than those measured with the blood gas analyzer (6.7 ± 1.3% with a range of 3.9–8.0%). No serious adverse events, cardiopulmonary dysfunction, or organ injury were reported, and mild symptoms such as headache or dizziness were transient and resolved without intervention.

### High-dose iNO in cystic fibrosis and nontuberculous mycobacteria patients

Numerous in vitro studies have shown that NO has dose-dependent bactericidal properties [31, 32], inhibits viral replication [33–36], and maybe synergistic with antibiotics such as amikacin and clofazimine against *Mycobacterium (M.) abscessus* [37]. Pre-clinical and clinical evidence suggests that iNO presents favorable clinical applications to treat lung infections due to its multiple mechanisms of action and unique properties—unlike common antibiotics, which typically rely on a single mechanism. NO induces DNA modifications such as deamination and cross-linking, disrupting microbial replication and repair mechanisms [19, 38]. It also inhibits aerobic respiration in pathogens like *Mycobacterium tuberculosis*, forcing dormancy [39], disrupts bacterial membranes [31, 32, 40], and depolarizes the cytoplasmic membrane in Gram-positive bacteria [31]. Beyond direct antimicrobial effects, NO disrupts biofilms formed by resistant organisms such as *P. aeruginosa* and *Klebsiella pneumoniae*, enhancing antibiotic efficacy and reducing bacterial shielding [41, 42]. Additionally, NO increases ciliary beat frequency via a cGMP-dependent pathway, improving mucociliary and bacterial clearance [43]. Recently, Okda et al*.* [14] summarized these mechanisms in a comprehensive review on the antimicrobial effects of NO.

Building on this mechanistic rationale, Deppisch et al*.* [44] were the first to investigate iNO in CF patients with chronic bacterial lung infections. They reported improved lung function (17.3% increase in FEV₁, P = 0.012), decreased airway inflammation, and reduced bacterial and fungal colonization, including *P. aeruginosa*, *M. abscessus*, and ESBL-producing *E. coli* (Table 2). Yaacoby-Bianu et al*.* [45] using intermittent 160 ppm iNO therapy found reduced sputum bacterial load and improved well-being in two CF patients with chronic *M. abscessus* infection. Bentur et al*.* [46] extended these findings in a broader safety and efficacy study, showing improvement in lung function and endurance, partial culture conversion, and reductions in bacterial DNA load, as quantified by qPCR. These early investigations indicated the biological activity of high-dose iNO in targeting drug-resistant infections while maintaining a favorable safety profile.

**Table 2:** High-dose iNO in cystic fibrosis and nontuberculous mycobacteria patients

Study	Study design	Patients	NO dose and source	Safety endpoint	Adverse event	Outcome
Deppisch et al. 2016	Phase I	8 patients with cystic fibrosis (CF) with chronic bacterial lung infections	160 ppm 30 min × 3/day for 2 periods of 5 days	SBP > 90 mmHg MetHb < 5% SaO₂ > 88% Pneumothorax or endotracheal hemorrhage Acute pulmonary exacerbations	4 subjects: transient xerostomia; 2 subjects: upper/lower respiratory tract infection with cough, not febrile	Reduction in colony-forming units of all bacteria and fungi 2. Improved lung function (FEV1), and reduced lung inflammation
			An 800 ppm NO/N₂ cylinder (Linde AG, Germany), was administered via an experimental NO-A device (Maquet GmbH, Germany)			
Yaacoby-Bianu et al. 2018	Prospective, open-label, compassionate use study	2 CF patients with persistent Mycobacterium abscessus infection	160 ppm intermittently (Patient 1: 26 days, Patient 2: 21 days; 30 min/session, ≥ 3.5 h apart)	SBP > 90 mmHg MetHb < 5% NO2 < 5 ppm SpO2 > 88%	0 subjects: reported adverse events	Reduced Mycobacterium abscessus load in sputum No adverse events; well-tolerated and potential CF infection adjuvant
			An 800 ppm NO cylinder balanced in nitrogen supplied by AIT Ltd			
Bentur et al. 2020	Pilot study	9 CF patients with refractory Mycobacterium abscessus infection	160 ppm × 30 min, 5/day for 14 days (inpatient) and 3/day for 7 days (ambulatory)	MetHb < 7% NO2 < 5 ppm SpO2 > 89% HR and BP Any adverse event incidence	1 subject: dizziness; 1 subject: dry mouth; 1 subject: hemoptysis; 1 subject: MetHb elevation over safety threshold (> 7%); 3 subjects: minor NO-related AEs; 1 subject: NO-related SAE (papilledema with blurred vision)	No culture conversion, but reduced bacterial load, delayed culture positivity 2. Improved FEV₁ and 6MWD
			An 800 ppm cylinder (AIT Ltd, N₂-balanced)			
Bogdanovski et al. 2020	Compassionate use	1 CF patient with pulmonary M. abscessus infection	160 ppm (5/day for 14 days, then 3/day for 7 days, 30 min/session), then 240 ppm for 8 days	MetHb < 10% NO2 < 3 ppm SpO2 desaturation Incidence of adverse events	Treatment course #1: 0 subjects: reported adverse events Treatment course #2: 1 subject: headache; 1 subject: anxiety; 1 subject: chest tightness/shortness of breath	Improved quality of life, lung function, and 6MWD No M. abscessus eradication 3. 240 ppm treatment stopped due to symptoms (no MetHb toxicity)
			NO from plasma arc device (Beyond Air, USA) filtered through NO₂ scrubber			
Goldbart et al. 2021	Case report	1 CF with Mycobacterium abscessus	iNO 150–250 ppm for 40 min, 4 × day for 4 weeks (hospital) and 2 × day for 2 weeks (ambulatory)	MetHb < 10% NO2 < 5 ppm SpO2 desaturation Hemodynamic instability (HR, RR, BP) Adverse event incidence	0 subjects: NO-related adverse events	Lung function: slight improvement in FEV₁/FVC ratio; increased 6MWT distance QoL: significant and sustained improvement in respiratory symptoms and vitality (day 93) Bacterial load: reduced mid-treatment, no complete eradication Chest CT (day 269): marked improvement in consolidation and infection
			The source of NO in this study was provided by Beyond Air (Rehovot, Israel and Garden City, USA)			
Bartley et al. 2020	Case report	1 CF patient with Burkholderia multivorans infection	160 ppm × 30 min x up to 3/day for 28 days	MetHb < 5% NO₂ < 1.5 ppm SpO₂ desaturation Hemodynamic instability (HR, RR, BP) Adverse event incidence	0 subjects: NO-related adverse events	Improved lung function Reduced pulmonary exacerbations Changed pathogen antibiotic resistance patterns
			The source of NO was an 850 ppm NO in a nitrogen tank supplied by (Airgas Inc., Radnor Township, Pennsylvania, USA)			
Flume et al. 2022	Open-label, proof-of-concept trial	10 adults with pulmonary nontuberculous mycobacterial disease (NTM-PD)	160 ppm × 50 min, 3/day, 5 days/week for 3 weeks	MetHb < 5% NO₂ < 3 ppm SpO2 desaturation Incidence of any adverse events	6 subjects: 26 AEs from mild to moderate; 1 subject: SAEs (transient ischemic attack)	Some microbiologic improvements observed, but not statistically significant Symptom scores improved without statistical significance Time-to-positivity suggested reduced bacterial burden in some participants
			Gas from 5000 ppm NO cylinder (Novoteris, LLC) delivered via INODD with NO2 ≤ 3 ppm			
Thomson et al. 2025	Pilot, open-label, single-arm and multicenter	15 nontuberculous mycobacteria patients	250 ppm inhaled NO, 40 min/session, 2–4 times daily (hospital then home) for 84 days, plus 90-day follow-up;	MetHb < 10% NO2 < 5 ppm The incidence of serious adverse events	9 subjects: experienced treatment-related adverse events, mainly mild (e.g., cough, hemoptysis, oropharyngeal pain, fatigue, hypotension, blurred vision, dry mouth, oral paresthesia, nausea, vomiting, headache, balance disorder, dysgeusia); no life-threatening events; no study device–related adverse events	14 of 15 patients completed treatment; 1 discontinued due to unrelated death Treatment well tolerated; 1 serious possible treatment-related adverse event (mild hemoptysis) Decreased bacterial load observed 4.One patient achieved culture conversion 5. Majority showed meaningful QoL improvements
			NO generated onsite from ambient air via LungFit® GO (Beyond Air, USA)			

High-dose iNO has also been used as a compassionate therapy in CF patients with no other viable treatment options, particularly for multidrug-resistant *M. abscessus* or *Burkholderia (B.) multivorans* infections. In three separate case reports, intermittent iNO was associated with improvements in lung function (FEV₁ and FVC), partial reductions in bacterial burden or improved antibiotic susceptibility, and significant gains in quality of life and functional capacity. In the case described by Bogdanovski et al*.* [47], iNO was used in a patient with *M. abscessus* infection and led to subjective respiratory improvement and small gains in FEV₁ and 6MWD. Goldbart et al*.* [48] reported more extensive benefits in a second patient with refractory *M. abscessus*, including sustained radiographic improvement, increased 6MWD, and transient reductions in bacterial load. Bartley et al*.* [49] described improved lung function, inflammatory marker reduction, and increased antibiotic susceptibility in a 16-year-old with *B. multivorans*, highlighting the potential for iNO to restore antibiotic sensitivity in resistant pathogens. In all cases, iNO treatment was well tolerated, with no serious adverse events reported.

Two studies have evaluated high-dose iNO therapy for refractory non-tuberculous mycobacterial pulmonary disease (NTM-PD). In an open-label, proof-of-concept trial, Flume et al*.* [50] evaluated the safety and feasibility of a 3-week course of iNO in adults with refractory NTM-PD across two centers in the US. While the study did not reach statistical significance in microbiological or symptom outcomes, 4 out of 10 patients achieved temporary culture conversion, and several reported improvements in cough and quality of life. Notably, the study demonstrated the technical feasibility and safety of high-dose iNO for this indication. Complementing these findings, Thomson et al*.* [51] conducted a Phase II pilot study in 15 patients with NTM-PD who had failed standard-of-care antibiotics. This study took the innovative step of assessing safety and efficacy of home-based iNO treatment utilizing a novel NO generator. Treatment compliance was > 90% across the 12-week treatment period. The study demonstrated a significant reduction in semiquantitative mycobacterial culture scores and improvements in respiratory and emotional well-being. One patient achieved sustained culture conversion, and only one SAE (mild hemoptysis) was possibly related to iNO therapy. These findings suggest antimicrobial activity of high-dose iNO and support its further evaluation in controlled trials, providing a preliminary foundation for evaluating the feasibility and safety of home-based administration in selected chronic infection settings.

Together, these studies highlight the need for multicenter clinical trials to further evaluate high-dose iNO as a potential, multimodal adjunctive strategy for patients with chronic, drug-resistant pulmonary infections, both in hospital and home settings.

### High-dose iNO in ARDS and SARS/COVID-19 patients

Nitric oxide targets the RNA-dependent RNA polymerase (RdRp), a critical enzyme for SARS-CoV-2 replication. RdRp function depends on iron–sulfur (Fe–S) clusters, which are disrupted by NO, thereby inhibiting viral RNA synthesis and replication [33]. This antiviral mechanism, distinct from traditional antivirals, inspired the testing of iNO as a potential therapeutic and prophylactic agent in respiratory viral infections, particularly COVID-19 [52].

Eleven clinical trials (Table 3) have evaluated iNO in patients with ARDS, COVID-19, or in perioperative settings for pneumonia prevention. These studies employed a range of delivery strategies, including intermittent high-dose and continuous low-dose iNO, and collectively explored iNO’s potential to modulate viral clearance, inflammation, clinical recovery, and postoperative complications.

**Table 3:** High-dose iNO in ARDS and COVID-19

Study	Study design	Patients	NO dose and source	Safety endpoint	Adverse event	Outcome
Chen et al. 2004	Rescue trial in Beijing	14 patients (6 treated) with SARS	30 ppm → 20 ppm → 10 ppm → 0 ppm over 4 days	Incidence of adverse events	0 subjects: NO-related adverse events	Improved arterial oxygenation Reduced need for mechanical ventilation and pressure support Decreased lung infiltrate density
			Delivered via INOmax/ INOvent (Datex-Ohmeda)			
Wiegand et al. 2020	Retrospective	5 patients with COVID-19	160 ppm × 30 min, 2/day for 3–5 days	MetHb < 5% NO_2_ < 2 ppm Changes in HR, RR, and BP SpO₂ desaturation Adverse effects incidence	0 subjects: NO-related adverse events	3 patients survived and were discharged 2 patients transitioned to comfort care and died
			NO from 850 ppm cylinder (Praxair, USA)			
Safaee et al. 2021	Phase I	29 Adults with COVID-19 (all treated)	160 ppm × 30 min, 2/day up to 14 days, until hospital discharge, negative SARS-CoV-2 rt-PCR, or 3 symptom-free days	MetHb < 5% NO_2_ < 5 ppm Pulmonary hypertension post-NO cessation Hypotension during NO therapy Acute kidney injury (AKI) development	0 subjects: NO-related adverse events reported	Decreased respiratory rate in tachypneic patients Improved oxygenation in hypoxemic patients 1 patient required intubation No deaths Median hospital stay: 6 days
			iNO from 850 ppm NO in N₂ cylinder (Praxair, USA)			
Safaee et al. 2020	Prospective	6 pregnant patients with severe bilateral COVID-19	160–200 ppm × 30 min, 2/day (2–18 treatments)	MetHb < 5% NO_2_ < 1.5 ppm SpO_2_ level HR and BP Incidence of AKI or adverse events	0 subjects: NO-related adverse events reported	Improved cardiopulmonary function Increased systemic oxygenation Reduced tachypnea during sessions No treatment-related adverse effects No maternal or neonatal deaths
			Continuous iNO < 40 ppm (Ventilated); NO from 850 ppm cylinder (Praxair, USA)			
Valsecchi et al. 2022	Phase I	71 pregnant patients with severe Bilateral COVID-19 (treated 20)	200 ppm × 30–60 min, 2/day for ~ 7 days until off oxygen	MetHb < 7% NO_2_ < 3 ppm HR and BP Serum creatinine levels Incidence of adverse events	0 subjects: NO-related adverse events reported	Improved respiratory function More oxygen-free days Shorter ICU and hospital stay No iNO-related maternal or neonatal adverse events
			NO from 850 ppm cylinder (Praxair, USA)			
Strickland et al., 2022	Prospective randomized	47 with acute respiratory symptoms enrolled; 34 SARS-CoV-2 + (19 treated) in ED	Single 250 ppm dose × 30 min	MetHb < 5% SpO₂ > 90% MAP↓ < 50 mm Hg	0 subjects; only transient, asymptomatic MetHb elevation managed by flow adjustment	No complications during administration MetHb levels normalized after discontinuation Low hospital readmission rate
			NO from 850 ppm cylinder (Airgas, USA)			
Pechyonkin et al. 2022	Pilot study	18 patients with moderate to severe COVID-19	1100 ppm for 5 min, 2–3/day for 5–10 days until ARDS signs resolved	MetHb ≤ 12% Total NO ≤ 12,000 ppm-min NO₂ ≤ 1,440 ppm-min	0 subjects; all patients survived and recovered, no NO-related complications reported	Immediate SpO₂ rise (e.g., 72% → 88%) Relief from distress and pain Faster lung recovery, ↓CRP, normalized coagulation No invasive ventilation needed All 18 survived, recovered, and discharged Better psychological state with improved breathing
			NO generated from air via PLASON device (Bauman Moscow State Technical University, Russia)			
Di Fenza et al. 2023	Phase II	193 (94 treated) Mechanically ventilated patients with COVID-19 pneumonia	Continuous iNO at 80 ppm for 48 h, then 40 ppm until PaO₂/FiO₂ > 300 mmHg	MetHb < 5% NO_2_ < 3 ppm Rebound hypotension or instability when reducing iNO Occurrence of AKI	0 subjects: NO-related adverse events reported	iNO improved oxygenation and PaO₂/FiO₂ ratio at 48 h Viral load reduction was faster and greater vs. control No difference in mortality or mechanical ventilation duration
			NO from iNO Therapeutics (MGH) and Linde plc (Dandery Hospital, Sweden)			
Kamenshchi-kov et al. 2024	Phase I	30 hypoxemic COVID-19 adults: 10 high iNO (200 ppm), 10 high + low iNO (200/20 ppm), 10 controls (SST)	200 ppm × 30 min, 2/day plus 20 ppm continuous; ~ 11 treatments per patient	MetHb < 5% NO_2_ < 3 ppm Hemodynamic instability (HR and BP) Incidence of adverse events or AKI	0 subjects: NO-related adverse events reported	Faster SARS-CoV-2 clearance with iNO (quicker rt-PCR negativity) Faster viral elimination did not correlate with clinical recovery speed No reduction in length of hospital stay
			NO from 2000 ppm tanks, controlled by Printer NOX analyzer (Care Fusion, USA)			
Wolak et al. 2024	Phase I/IIa	35 Adults with COVID-19 (treated 16)	150 ppm × 40 min, 4/day for up to 7 days based on response	MetHb < 10% NO_2_ < 5 ppm SpO_2_ > 89% Hemodynamic instability (BP, HR, RR) Adverse event incidence	2 subjects: iNO-related hypoxia, bradycardia	More treatment subjects reached SpO₂ ≥ 93% during hospitalization iNO group required fewer days of oxygen therapy
			NO generated from ambient air by LungFit^®^ PRO (Beyond Air, USA) with NO_2_ filter			
Friedrich et al. 2025	Phase I, open-label, parallel, randomized controlled trial​	110 COVID-19 patients (treated 55)	160 ppm for 6 h within first 48 h, single/fractional dose	MetHb < 7% NO_2_ < 5 ppm SpO_2_ > 92% Incidence of adverse events	1 subject: mild discomfort after 3 h of iNO inhalation (non-serious, not associated with MetHb or SaO_2_ changes) 0 subjects: NO-related adverse events	iNO group had more oxygen-free days vs. control (*p* = 0.044) Shorter hospital stay in iNO group (*p* = 0.004) Better clinical severity scores in iNO group on days 3 (*p* = 0.010) and 5 (*p* = 0.033) More patients weaned off ventilatory support by day 3 in iNO group (*p* = 0.005) No significant differences in mortality, severe disease progression, or SARS-CoV-2 PCR positivity at day 5
			NO (500 ppm in N₂) from medical-grade cylinders, regulated by custom digital device; continuous NO/NO₂ monitoring via NOx G Series			

The first signal came during the 2002 SARS outbreak, when Chen et al*.* [53] observed that iNO not only improved oxygenation but also led to persistent radiographic improvements. This led to the first clinical hypothesis that iNO might exert direct antiviral effects, moving beyond the conventional role of improving ventilation–perfusion matching.

Wiegand et al*.* [54] were among the first to explore the feasibility of high-dose iNO as a rescue therapy in non-intubated, spontaneously breathing COVID-19 patients. Conducted at Massachusetts General Hospital (Boston, MA), this case series introduced the concept of administering repeated intermittent iNO sessions outside of the ICU, demonstrating not only tolerability but also subjective symptomatic relief and hospital discharge in patients with early disease. Importantly, this study expanded the application of iNO beyond ventilated patients and offered a foundation for early-intervention use. Safaee et al*.* [55] advanced the field by conducting a larger feasibility trial in non-intubated COVID-19 patients, providing prospective evidence of clinical improvement, including reduction in respiratory rate and viral clearance within 28 days in 70% of cases. This was the first study to systematically link high-dose iNO to potential antiviral activity in a non-intubated population, supporting the hypothesis that NO may influence disease course beyond its effects on oxygenation.

Safaee et al*.* [56] conducted the first study of high-dose iNO in pregnant patients with COVID-19, at a time when no approved therapies were available for this population due to safety concerns. This pioneering prospective case series demonstrated that iNO was feasible, well-tolerated, and associated with immediate symptomatic relief in both ICU and non-ICU patients. Importantly, the study provided initial evidence of maternal and fetal safety, including favorable delivery outcomes and neonatal SARS-CoV-2 negativity. By initiating this work during a period of therapeutic uncertainty for pregnant individuals, Dr. Safaee’s study laid the groundwork for the safe inclusion of pregnant patients in future respiratory therapeutic trials. Valsecchi et al*.* [57] followed with the largest multicenter study to date of pregnant patients with severe COVID-19 treated with iNO, confirming not only favorable maternal outcomes, including more oxygen-free days and shorter hospitalization, but also comparable neonatal outcomes to standard care. This retrospective cohort study further validated the safety and translational potential of high-dose iNO in obstetric critical care and built upon the initial feasibility findings from Safaee et al*.*

Strickland et al*.* [58] conducted a prospective randomized trial testing the practicality of administering a single session of high-dose iNO in the Emergency Department (ED). This was the first randomized study targeting early COVID-19 in spontaneously breathing patients, showing that iNO at 250–300 ppm was well-tolerated in the ED setting. Though prematurely terminated due to declining case numbers, this trial opened new frontiers for pre-hospital or early hospital-based use of antimicrobial gas therapies.

In Russia, Pechyonkin et al*.* [59] tested ultra-high-dose iNO (1100 ppm) in severely hypoxemic patients, reporting full survival and resolution of inflammation without need for mechanical ventilation. Meanwhile, Di Fenza et al*.* [60] conducted a multicenter randomized trial in intubated COVID-19 patients, showing 80 ppm iNO accelerated viral clearance, reduced neurological symptoms at 90 days, and transient improvements in gas exchange. Although mortality was unaffected, the trial provided valuable safety and translational data for use in critically ill patients.

Further refining delivery strategies, Kamenshchikov et al*.* [61] combined intermittent 200 ppm with continuous 20 ppm in spontaneously breathing patients, demonstrating short-term gains in oxygenation and lung function, with acceptable safety. Wolak et al*.* [62] corroborated these results in a multicenter open-label trial, showing shorter oxygen support duration and faster stabilization in patients with viral pneumonia.

Friedrich et al*.* [63] performed a randomized Phase I trial in hospitalized adults with moderate COVID-19, reporting that iNO led to significantly more oxygen-free days, shorter hospital stay, and faster clinical improvement. This was the first trial to demonstrate functional recovery endpoints in COVID-19 patients treated with iNO.

Despite the promising results from the aforementioned trials, larger Phase III studies are warranted to further evaluate the efficacy and long-term antimicrobial effects of high-dose iNO across diverse populations.

### High-dose iNO in cardiac surgery

Beyond COVID-19, Kalashnikova et al*.* [64] conducted a novel prospective RCT in moderate-risk patients undergoing elective cardiac surgery (Table 4). They demonstrated that prophylactic iNO significantly reduced postoperative pneumonia and preserved spirometry parameters, positioning iNO as a potential preventive agent in surgical patients. Kamenshchikov et al*.* [65] further supported these findings in a recent parallel cardiac surgery trial, where pneumonia incidence was halved (14.7% vs. 29.4%) and iNO remained well tolerated. These studies suggest a promising new role for NO-based therapy in perioperative infectious risk management.

**Table 4:** High-dose iNO in cardiac surgery

Study	Study design	Patients	NO dose and source	Safety endpoint	Adverse event	Outcome
Kalashnikova et al. 2025	Pilot study	74 elective cardiac surgery patients at pneumonia risk; 37 received iNO	200 ppm × 30 min, 2/day for 5 days	MetHb < 5% NO_2_ < 3 ppm Hemodynamic instability (BP, HR, RR) Postoperative pneumonia incidence during hospital stay	0 subjects: NO-related adverse events reported	1. iNO reduced postoperative nosocomial pneumonia incidence vs. control (*p* = 0.046) 2. No increase in acute kidney injury incidence with iNO
			NO from plasma-chemical device (Tianox KS, Russia)			
Kamenshchi-kov et al. 2025	Randomized, controlled trial	136 patients with CKD undergoing elective cardiac surgery with CPB	80 ppm × intraop + 6 h postop, single course	MetHb < 5% NO_2_ < 3 ppm Blood transfusion frequency, blood loss volume, platelet count (POD1) Oxidative/nitrosyl stress markers	0 subjects: NO-related adverse events reported	1. ↓ AKI incidence with iNO (23.5% vs. 39.7%, *p* = 0.043) 2. ↑ eGFR at 6 months (*p* = 0.038) 3. ↓ postoperative pneumonia (*p* = 0.039) 4. No safety concerns (MetHb, NO₂, bleeding, oxidative stress)
			NO (80 ppm) from plasma-chemical device (Tianox KS, Russia)			

### High-dose iNO in bronchiolitis

The use of high-dose iNO as an adjunctive therapy in infants with acute bronchiolitis has emerged as an innovative approach to treating a viral illness that currently lacks effective pharmacologic therapies. A series of three randomized controlled trials in Israel have systematically advanced the field by establishing the feasibility, safety, and dose-dependent efficacy of intermittent high-dose iNO in this vulnerable population (Table 5). Tal et al*.* [66] conducted the first pilot randomized controlled trial to test high-dose iNO in hospitalized infants with moderate bronchiolitis. While the primary outcome showed no group difference, a post hoc analysis revealed significantly shorter hospital stays in infants hospitalized for more than 24 h who received iNO. This was the first demonstration that iNO could meaningfully impact clinical recovery in selected subgroups of infants with viral bronchiolitis. Building on these findings, Goldbart et al*.* [67] conducted a multicenter double-blind RCT and demonstrated that iNO-treated infants with acute bronchiolitis had a significantly shorter length of stay and achieved target oxygen saturation levels earlier than controls. This trial helped to verify the concept that iNO could accelerate clinical stabilization in bronchiolitis through mechanisms potentially related to its antiviral and anti-inflammatory effects. In a follow-up dose-finding RCT, Goldbart A et al*.* [68] compared two iNO doses (150 ppm and 85 ppm) against standard therapy. The 150 ppm group showed superior outcomes across multiple endpoints, including faster time to discharge and quicker oxygen stabilization, with no increase in treatment-related serious adverse events. This was the first pediatric RCT to define an optimal therapeutic dose of high-dose iNO, providing a critical reference point for future trials.

**Table 5:** High-dose iNO in bronchiolitis

Study	Study design	Patients	NO dose and source	Safety endpoint	Adverse event	Outcome
Tal et al. 2018	Pilot, randomized, double-blinded, controlled trial	43 Infants with acute bronchiolitis (treated 21)	160 ppm × 30 min, 5/day for up to 5 days	MetHb < 5% NO₂ < 5 ppm Bleeding episodes Adverse events	5 subjects: iNO-related AEs; 4 subjects: iNO-related SAEs	No overall length of stay difference between groups In infants hospitalized > 24 h, NO group had shorter median stay (41.9 vs. 62.5 h, *p *= 0.014)
			NO from 800 ppm cylinders (Maxima Medical, Israel)			
Goldbart et al. 2020	Pilot study	69 infants with acute bronchiolitis (34 treated)	160 ppm × 30 min, 5/day for up to 5 days	MetHb < 7% NO₂ < 5 ppm Adverse events	13 infants: transient O₂ desaturation (80 − 88%, ≤ 30 s) during iNO inhalation	↓ length of stay Rapid oxygen saturation improvement No adverse events vs. control
			NO from 800 ppm cylinder balanced in nitrogen (Maxima Medical/Gordon Gas)			
Goldbart et al. 2023	Phase I/IIa	89 Infants with severe bronchiolitis (treated 29 with 150 ppm vs 32 with 85 ppm vs 26 controls)	150 ppm × 40 min × 4/day vs. 85 ppm × 40 min × 4/day, up to 5 days	MetHb < 7% NO₂ < 3 ppm HR and RR changes Adverse events	0 subjects: NO-related adverse events reported	150 ppm iNO reduced time to fit for discharge and hospital discharge No significant efficacy difference between 85 ppm iNO and control
			NO from 800 ppm cylinder balanced in nitrogen (Maxima Medical/Gordon Gas/Oxygen and Argon Works)			

These trials represent a novel body of work demonstrating that high-dose iNO is not only safe and well tolerated in infants, but may also reduce hospitalization time and improve clinical recovery in bronchiolitis, a field where effective antiviral interventions are urgently needed. The findings lay the foundation for larger, multicenter international studies and raise the possibility of using iNO as a disease-modifying therapy in other forms of pediatric viral pneumonia.

Together, these studies illustrate the evolution of high-dose iNO from a physiological gas used for oxygenation to a multifunctional therapeutic agent with direct antiviral, antibacterial, and immunomodulatory effects. From its initial application during the SARS outbreak to its use in COVID-19 patients, including infants, pregnant individuals, outpatients, and emergency department presentations, iNO has demonstrated a consistent safety profile and promising signals of clinical benefit. Importantly, the transition from ICU-based delivery to home and perioperative use marks a significant step toward broader access and real-world feasibility. These findings support the continued investigation of iNO as a novel adjunctive or primary therapy for viral pneumonia, drug-resistant lung infections, and postoperative respiratory complications, and they underscore its potential to fill critical gaps where conventional therapies fall short.

## Conclusions and future directions

High-dose iNO is emerging as a potential therapeutic platform that extends far beyond its traditional role as a selective pulmonary vasodilator. Across a diverse body of clinical and translational studies, high-dose iNO has demonstrated a unique constellation of effects, including direct antimicrobial activity, inhibition of viral replication, disruption of biofilms, reduction of inflammation, and enhancement of mucociliary clearance. These properties position iNO as a versatile agent capable of addressing urgent clinical needs in both acute care and chronic infection management.

## Limitations and safety considerations

Safety considerations are particularly relevant when repurposing iNO at supraphysiologic doses for antimicrobial indications. Indeed, despite these advances, several critical gaps remain. Current delivery systems for NO rely on bulky and costly gas cylinders, which increase public healthcare costs and limit its use in certain clinical environments and with controlled delivery platforms. Emerging technologies, such as electrically generated high-dose NO, offer the potential to enable more portable, cost-effective delivery, making its use feasible even in low-resource settings [69, 70]. The optimal dose, frequency, and duration of therapy remain poorly defined and appear to be disease-specific. In bronchiolitis, a dose of 150 ppm was superior to lower doses; in NTM lung disease, intermittent high-dose regimens based on in vitro activity have shown benefit. In ARDS and COVID-19 patients, trials have tested a wide range of regimens with variable efficacy outcomes, suggesting that a more mechanistically grounded approach to dose selection is needed.

Equally important is the standardization of safety monitoring. It has been established that iNO inhibits human platelet aggregation in vitro and in vivo [71, 72]. In contrast, several prospective randomized controlled trials reported that iNO did not affect platelet counts and coagulation function in newborns [73] and in adults [30, 65, 74]. Thus, carefully monitoring platelet and coagulation function is important during iNO delivery. While most studies have adopted MetHb thresholds of 5%-10% and NO_2_ limits of 3–5 ppm, there is no consensus on when to hold or adjust therapy, and some trials have used real-time monitoring while others relied on post hoc lab checks. The U.S. Occupational Safety and Health Administration (OSHA) 8-h permissible exposure limit (PEL) for NO_2_ is 5 ppm. Higher MetHb levels have been observed in specific indigenous populations than the general population, particularly among Alaskan Natives, Athabaskan Indians, Navajo Indians, and the Evenk people of Yakutia [75–77]. This is not a universal trait among all indigenous peoples but is linked to genetic enzyme deficiencies that are more prevalent in these isolated communities. Genetic deficiency is a primary cause in some indigenous groups, including enzyme deficiency (glucose-6-phosphate dehydrogenase (G6DP) and cytochrome b5 reductase (CYB5R)), inheritance pattern (an autosomal recessive trait), specific gene (CYB5R3), and affected populations (some indigenous populations and some high-altitude natives). Environmental influence, such as dietary intake, is another factor causing methemoglobinemia. However, those people with high baseline levels of MetHb are often asymptomatic. Therefore, in certain populations with high baseline MetHb levels, caution should be taken during iNO treatment, particularly regarding changes in MetHb levels before and after iNO administration, rather than the absolute MetHb values. Harmonized thresholds, real-time monitoring protocols, and contingency plans for treatment interruption must become standard in future multicenter trials.

Another safety concern is the global impact of NO on immune cells. NO is a key immunoregulatory molecule and plays a dual role in the immune system, acting as both a protective agent and a destructive one, with its impact depending on concentration and cell type. NO modulates immune cell function, including the proliferation, differentiation, and death of lymphocytes, macrophages, and neutrophils [78]. It has substantial potential for modulating immune dysregulation in various diseases, such as cancer, rheumatoid arthritis, osteoarthritis, peritonitis, neuroinflammation, inflammatory bowel disease, infectious diseases, and wound healing. These examples demonstrate the use of NO-modulating systems for fine-tuning immune responses in a disease-specific manner. Although NO eradicates bacteria and viruses in vitro and in vivo models, its effects on host immune training, antigen presentation, and immune memory formation remain largely unexplored in infectious disease settings.

In addition, the possible risks of iNO to eukaryotic human cells, as well as its potential involvement in cancer development or progression, warrant further long-term studies to ensure safety and clinical applicability.

In chronic infection settings, including CF and NTM-PD, there is growing interest in home-based high-dose iNO therapy. Portable electrochemical generators now make this feasible [51, 79, 80]. However, rigorous clinical trials are needed to validate efficacy and define appropriate delivery protocols, including whether iNO should be administered alone or in combination with antibiotics, and how to measure microbiological response over time.

In parallel with clinical work, basic and translational studies are urgently needed to define microbial susceptibility to NO, potentially synergy or antagonism with antibiotic therapy, its interaction with the host immune system, and the pharmacokinetics of repeated high-dose inhalations. These studies should inform clinical trial design, ideally enabling a precision medicine approach to iNO therapy.

## Future directions and research roadmap

Recognizing these limitations and safety considerations, the next phase of investigation must focus on carefully designed translational and clinical studies to clarify the therapeutic potential of high-dose inhaled nitric oxide. Specifically, the range of potential indications for high-dose iNO is likely underappreciated. Conditions such as ventilator-associated pneumonia, hospital-acquired pneumonia, tuberculosis, lung transplantation, post-viral fibrosis, and infections in the immune-suppressed host, represent fertile ground for future research. In each, the combination of antimicrobial action, immune modulation, and mucociliary enhancement offers a mechanistic rationale worthy of investigation (Table 6).

**Table 6:** Roadmap for advancing inhaled nitric oxide as antimicrobial therapy

Clinical and research domains	Key actions and objectives
Dosing and regimen optimization	Define condition-specific dose ranges (e.g., 150–300 ppm), frequency, and duration for effective therapy
Standardized safety monitoring	Implement real-time monitoring of methemoglobin (MetHb) and nitrogen dioxide (NO₂) with clear thresholds
Efficacy endpoints by indication	Tailor primary outcomes by disease: e.g., culture conversion, time to discharge, and viral load reduction
Home-based therapy protocols	Develop and validate chronic-use protocols using portable delivery systems for non-hospitalized patients
Parallel mechanistic research	Investigate pharmacokinetics, microbial susceptibility, and immune modulation in conjunction with clinical trials
Expansion to new indications	Evaluate efficacy in ventilator-associated pneumonia (VAP), hospital-acquired pneumonia (HAP), tuberculosis (TB), immunocompromised hosts, and post-viral fibrosis
Phase III clinical trials	Conduct multicenter outcome trials in selected high-need patient populations to confirm safety and efficacy

The field now stands at a pivotal juncture. With early-phase safety established and delivery systems improving, the next step is the execution of well-designed, adequately powered phase III trials, alongside a coordinated research agenda that includes mechanistic science, microbiology, and device innovation. If pursued with scientific rigor, high-dose iNO might have the potential to become a transformative therapy in respiratory medicine, addressing not just oxygenation, but infection, inflammation, and recovery.

## Data Availability

Not applicable.
